# Optical Properties and Growth Mechanism of La_3_Ga_5.5_Nb_0.5_O_14_ Macroporous Ceramic

**DOI:** 10.1186/s11671-019-3104-x

**Published:** 2019-08-16

**Authors:** Yu-Jen Hsiao, Yempati Nagarjuna, Sheng-Chang Wang

**Affiliations:** Department of Mechanical Engineering, Southern Taiwan University of Science and Technology, Tainan, 710 Taiwan

**Keywords:** La_3_Ga_5.5_Nb_0.5_O_14_, Macroporous, Optical properties, Pechini process

## Abstract

Optical properties and growth mechanism of La_3_Ga_5.5_Nb_0.5_O_14_ oxides by a Pechini process were investigated. The structure and morphology were obtained after sintering at 600–800 °C. This crystallized orthorhombic La_3_Ga_5.5_Nb_0.5_O_14_ can be obtained by the heat treatment process at 800 °C from XRD. A proposed schematic growth mechanism of La3Ga5.5Nb0.5O14 macroporous based on the details provided is shown. The photo-luminescence spectra shown that under 327 nm excitation spectra, a broad and blue emission peak is observed at 475 nm at 77 K and this spectrum is originated from the [NbO_6_]^7−^ octahedra group. The optical absorption spectra of the 800 °C sample exhibited a well-crystalline and very low oxygen vacancy, which corresponded to the band gap energies of 3.95 eV.

## Introduction

The piezoelectric and optical properties of Ca_3_Ga_2_Ge_4_O_14_ (CGG)-type structure of La_3_Ga_5_SiO_14_ (LGS) [1], La_3_Ga_5.5_Nb_0.5_O_14_ (LGN) [2], and La_3_Ga_5.5_Ta_0.5_O_14_ (LGT) [3] compounds have been actively and systematically studied. They have been investigated for bulk acoustic wave (BAW) and surface acoustic wave (SAW) devices for the fabrication of filters with large pass bandwidths and oscillators with a large shift or high-frequency stability [4–7]. The piezoelectric and dielectric oxide has been applied in the optical field [8, 9]. A promising nonlinear oxide crystal La_3_Ga_5.5_Nb_0.5_O_14_ (LGN) is proposed and fully characterized in recent years. The mid-infrared spectral range extending from 2 to 6 μm is significant for scientific and technological applications [10]. The phase-matching angles of second-harmonic generation and difference-frequency generation up to 6.5 μm was also measured in the langanate crystal La_3_Ga_5.5_Nb_0.5_O_14_ (LGN) [11]. Voda et al. [12] reported that it was used both as a laser and a laser host material. Nd:La_3_Ga_5.5_Nb_0.5_O_14_ laser properties of output power and wavelength temperature tuning under laser diode pumping are also verified [13].

Macroporous materials have porosity ranging from 5 to 90% and pore size ranging more than 100 nm. Macroporous materials have diverse properties like excellent mechanical strength, high thermal conductivity, good chemical resistance, and high thermal shock resistance which led into the industrial applications like filtration for water and gas, thermoelectric convertor, catalytic agent [14]. Due to photonic band gaps, macroporous photonic crystals have been used for advanced applications such as optical communications, light emissions, and gas sensing. Macroporous photonic crystals of different materials can be used in chemical detections, and this led to explore different macroporous materials for gas sensing. Porous ceramics are of great interest due to their numerous potential applications in industries such as catalysis, adsorption and separation, filtration of molten metals or hot gases, refractory insulation of furnaces, and hard tissue repair and engineering [15].

Recently, very few works have been done on LGN-based macroporous ceramic formed by the chemical method. Yu [16] prepared piezoelectric crystal of La_3_Ga_5.5_Nb_0.5_O_14_ by using the sol-gel process and examined microstructure analysis. The results have shown that LGN nanoparticles were crystallized in the trigonal crystallographic phase. Kong [17] studied the growth of single-crystal La_3_Ga_5.5_Nb_0.5_O_14_ (LGN) by the Czochralski method which requires high temperature such as more than 1500 °C. In our study, macroporous polycrystal was developed by using the Pechini process which requires low temperature such as 800 °C. The polycrystal and the single crystal have the same orthorhombic phase. The main purpose of this work is to use a Pechini process for the preparation of a single-phase La_3_Ga_5.5_Nb_0.5_O_14_ LGN ceramic with the calciumgallogermanate (CGC-type) structure (trigonal, space group P321) [18]. The major advantage of the Pechini process is that there is a low-temperature processing [19–21]. Chemically synthesized ceramic powders often possess better chemical homogeneity and better size control of particle morphology than those produced by the mixed oxide route [22]. Therefore, the optical properties and growth mechanism of LGN macroporous oxide have been investigated in this study.

## Methods/Experimental

### Materials Used

Lanthanum nitrate La(NO_3_)_3_, gallium nitrate Ga(NO_3_)_3_, niobium chloride (NbCl_5_), citric acid anhydrous (CA), and ethylene glycol (EG).

### Preparation of La_3_Ga_5.5_Nb_0.5_O_14_

The La_3_Ga_5.5_Nb_0.5_O_14_ macroporous ceramics were prepared by the Pechini process using lanthanum nitrate La(NO_3_)_3_, gallium nitrate Ga(NO_3_)_3_, niobium chloride (NbCl_5_), citric acid anhydrous (CA), and ethylene glycol (EG). All materials have over 99.9% purities. According to the reaction, niobium ethoxide, Nb(OC_2_H_5_)_5_ synthesis takes place from niobium chloride NbCl_5_ and ethanol, C_2_H_5_OH.

1 $$ {\mathrm{NbCl}}_5+5{\mathrm{C}}_2{\mathrm{H}}_5\mathrm{OH}\to \kern0.5em \mathrm{Nb}{\left({\mathrm{OC}}_2{\mathrm{H}}_5\right)}_5+5\mathrm{HCl} $$

The stoichiometric amount of lanthanum nitrate, gallium nitrate, and niobium ethoxide was dissolved in water. A chelating agent such as citric acid is added to the solution. The molar ratio of citric acid and metal ions used in this process is 2:1. A stabilizing agent such as ethylene glycol is added to the above solution. The precursor containing La, Ga, and Nb were dried in an oven at 120 °C for 24 h, and then, the La_3_Ga_5.5_Nb_0.5_O_14_ ceramics were obtained after sintering at 600–800 °C for 3 h in air.

### Characterization/Phase Identification

The burnout behaviors of powders were analyzed by differential thermal analysis and thermogravimetry analysis (DTA–TGA, PE–DMA 7). The phase identification was performed by X-ray powder diffraction (Rigaku Dmax-33). The morphology and microstructure were examined by transmission electron microscopy (HR-TEM, HF-2000, Hitachi). The excitation and emission spectra were recorded on a Hitachi-4500 fluorescence spectrophotometer equipped with xenon lamp at 300 K and 77 K. The absorption spectra were measured using a Hitachi U-3010 UV–vis spectrophotometer at room temperature.

## Results and Discussion

The amorphous compound is subjected to heat treatment to undergo pyrolysis process to become crystalline structure. In this experiment, the possible chemical reactions for the synthesis of La_3_Ga_5.5_Nb_0.5_O_3_ powders can be expressed as follows:

2 $$ {\displaystyle \begin{array}{l}\kern1.56em 3\mathrm{La}{\left({\mathrm{NO}}_3\right)}_3+5.5\mathrm{Ga}\left(\mathrm{NO}3\right)2+0.5\mathrm{Nb}{\left({\mathrm{O}\mathrm{C}}_2{\mathrm{H}}_5\right)}_5\overset{\mathrm{C}\mathrm{A}}{\to}\\ {}\to {\mathrm{La}}_3{\mathrm{Ga}}_{5.5}{\mathrm{Nb}}_{0.5}{\mathrm{O}}_3+{\mathrm{NO}}_2\uparrow +{\mathrm{H}}_2\mathrm{O}\uparrow +{\mathrm{C}\mathrm{O}}_2\uparrow +{\mathrm{C}}_2{\mathrm{H}}_5\mathrm{OH}\uparrow \end{array}} $$

**Fig. 1:**
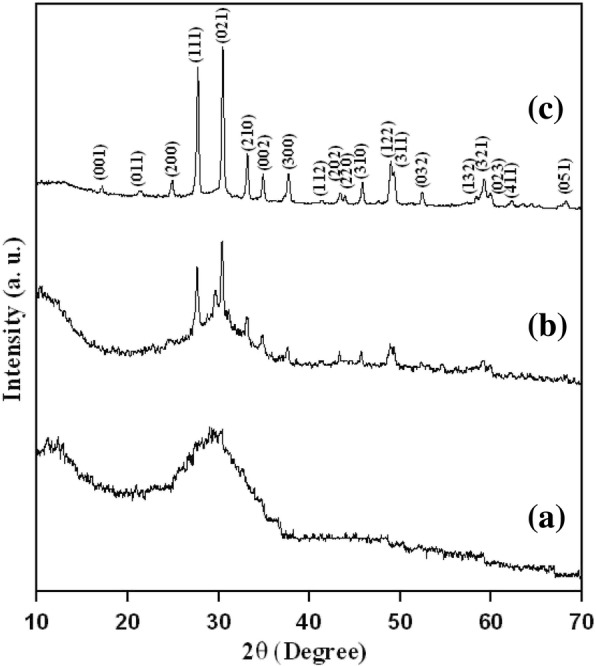
X-ray diffraction patterns of La_3_Ga_5.5_Nb_0.5_O_14_ precursor powders annealed at (**a**) 600, (**b**) 700, and (**c**) 800°C for 3 h

This precursor powder is heat-treated for about 3 h at 600–800 °C, and the XRD patterns for this temperature are shown in Fig. 1. At 600 °C calcined temperature, the precursor powder has shown its small amount of microcrystal structure. When the temperature raised to 700 °C, decomposition of amorphous powder takes place and it begins to crystallize. When the sintered temperature reaches 800 °C, the precursor powder sample is shown in a single phase which is orthorhombic La_3_Ga_5.5_Nb_0.5_O_14_ phase (JCPDS file no. 47-0533) where the peaks are identified. This sharper peak shows that the crystalline form of the La_3_Ga_5.5_Nb_0.5_O_14_ powder. With the increase in temperature, the intensity of the peaks becomes sharper which indicates the crystalline structure of the La_3_Ga_5.5_Nb_0.5_O_14_ powder.

**Fig. 2:**
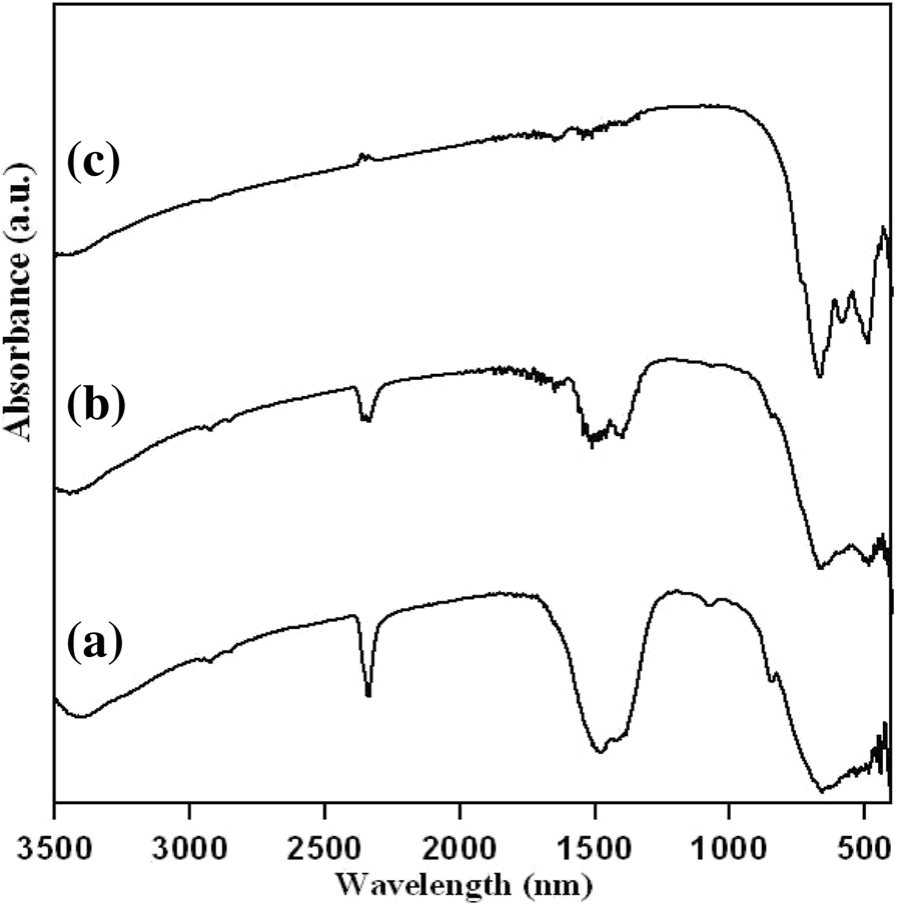
FT–IR spectra of dried powders at (**a**) 600, (**b**) 700, and (**c**) 800 °C for 3 h with a composition of La_3_Ga_5.5_Nb_0.5_O_14_

The FT-IR spectra of the powder at 600–800 °C are shown in Fig. 2. Figure 2a, b shows the IR spectra of powder at 600 to 700 °C, respectively, where there is a sharp stretch at 2300 nm wavelength which identifies the presence of strong carbon dioxide compound class stretching and there is a bulk stretch at 1500 nm wavelength which indicates the absorbed nitrate ions in the structure [14]. So, these might be the strong stretching vibrations of nitrate ions. From Fig. 2c, there are new peaks formed at 500 to 600 nm when the annealing temperature is increased to 800 °C. This new peak indicates the formation of the La_3_Ga_5.5_Nb_0.5_O_14_ nanocrystals. The peaks present at 1500 nm and 2300 nm wavelength are calcined at 800 °C. This shows the presence of little residues in the organic compound.

**Fig. 3:**
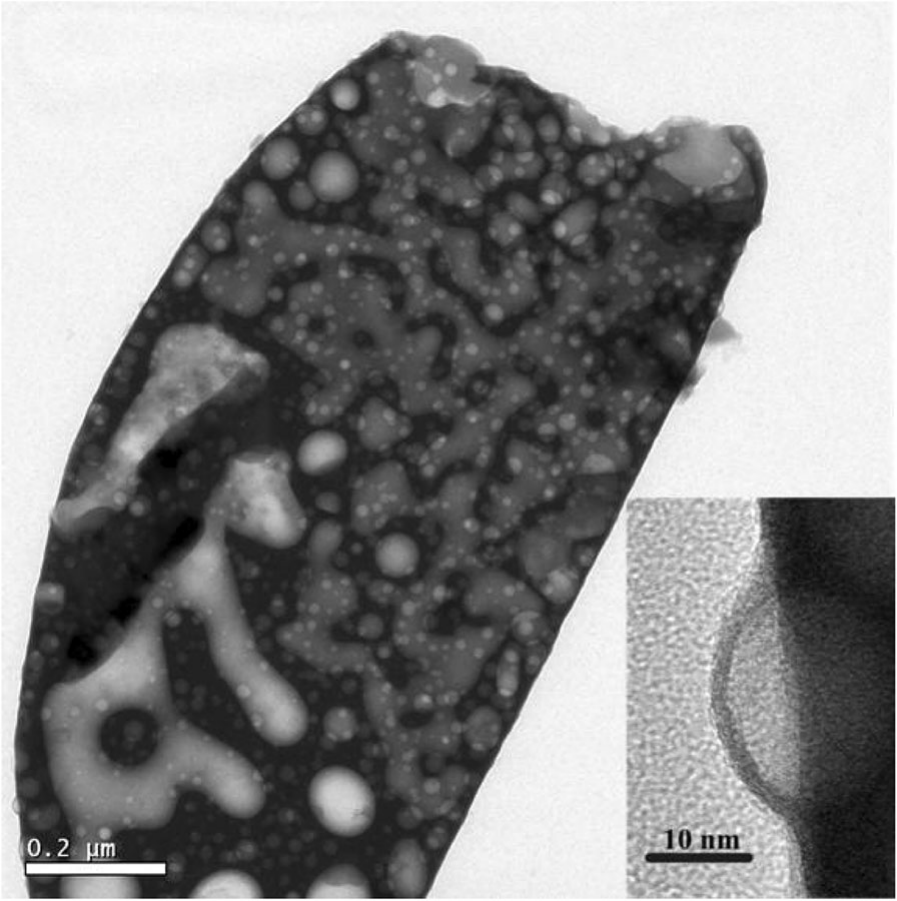
The morphology of as-synthesized La_3_Ga_5.5_Nb_0.5_O_14_ at 600 °C. The inset is the TEM images of nanofoam at the precursor’s surface

TEM images of the precursor powder at 600 °C for the morphology are shown in Fig. 3. The first image shows the size and shape morphology of the precursor ceramic. From the figure, different sizes of nanofoams are present in the precursor powder. The second image is the enlarged version and side view of the first image. It is shown that bulged nanofoams are present on the surface of the precursor at the beginning of crystal formation. These nanofoams are of different sizes as seen from the image. These nanofoams have a low thickness, and when the temperature increases, these nanofoams form microporous holes. This shows the particles have a semi-circular shape which is hollow inside. This nanoporous material is a nanofoam which contains gas inside of it. When the temperature will increase, these nanofoams will leave a hole of diameter less than 100 nm.

**Fig. 4:**
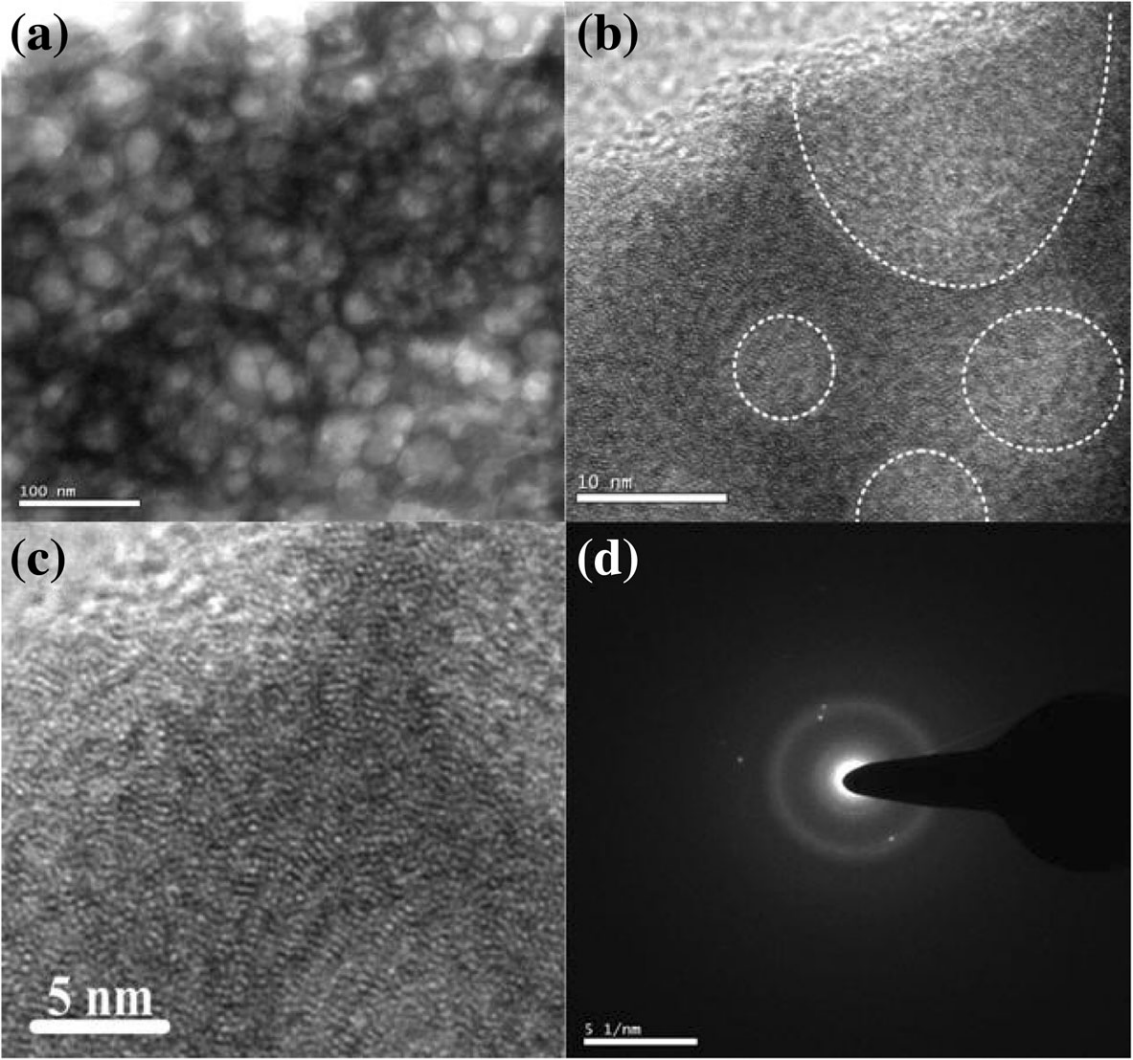
**a** TEM images of as-synthesized La_3_Ga_5.5_Nb_0.5_O_14_ at 700 °C, **b** high-resolution TEM image of the microporous, **c** the lattice image for short-range order nanocrystalline structure, and **d** electron diffraction pattern of the short-range order nanocrystalline area

TEM images of different magnification is showed in Fig. 4. In the Fig. 4(a), the magnification is very low which is 100 nm and the structure visibility is not good. In Fig. 4(b) the TEM image is magnified to 10 nm and crystal structure can be seen. In Fig. 4(c) image is enlarged to 5 nm. Fig. 4(d) represents the small amount of micro crystal structure.

**Fig. 5:**
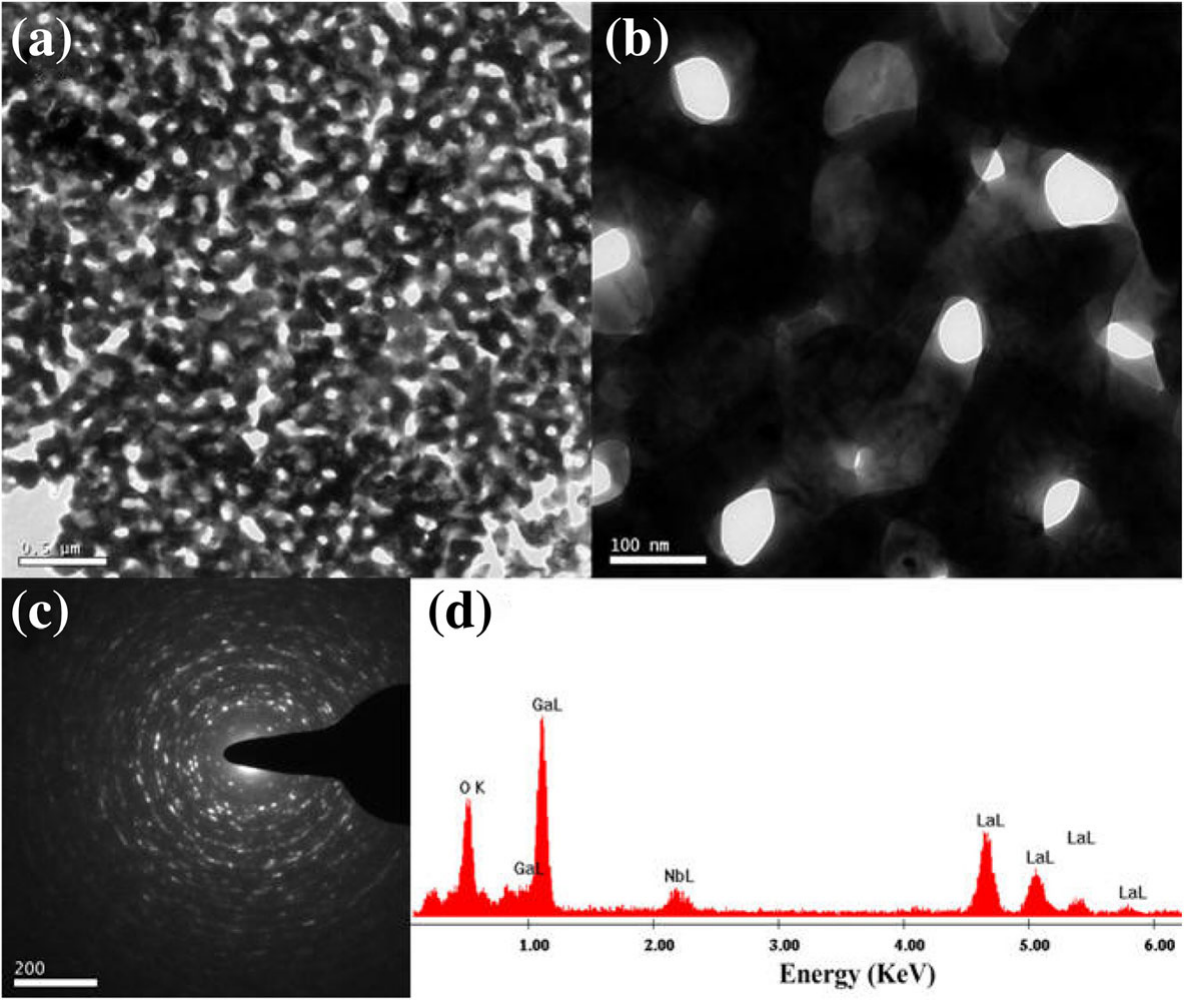
**a** TEM images of as-synthesized macroporous at 800 °C, **b** high-resolution TEM image of the macroporous, **c** electron diffraction pattern, and **d** EDX analysis of La_3_Ga_5.5_Nb_0.5_O_14_ macroporous oxide

TEM analysis for the crystal at 800 °C is shown in Fig. 5. The first Fig. 5a magnification is very low which shows the nanocomposition of the crystal form. Figure 5b is a highly magnified image of the crystal where the structure of the macroporous crystal is seen. The bright images are the air holes which are formed from the nanofoams. Electron diffraction of the La_3_Ga_5.5_Nb_0.5_O_14_ crystalline structure is shown in Fig. 5c. There are circular bright continuous rings in the electron diffraction pattern. This indicates that the particles were nanosized, and it also confirms the crystalline nature of the nanoparticles [23]. EDX analysis of La_3_Ga_5.5_Nb_0.5_O_14_ macroporous oxide is shown in Fig. 5d. This analysis shows about the molar ratio of the structure La_3_Ga_5.5_Nb_0.5_O_14_. The peak of Ga is high which indicates it has more content. Nb has a very low peak since it is very less in content.

**Fig. 6:**
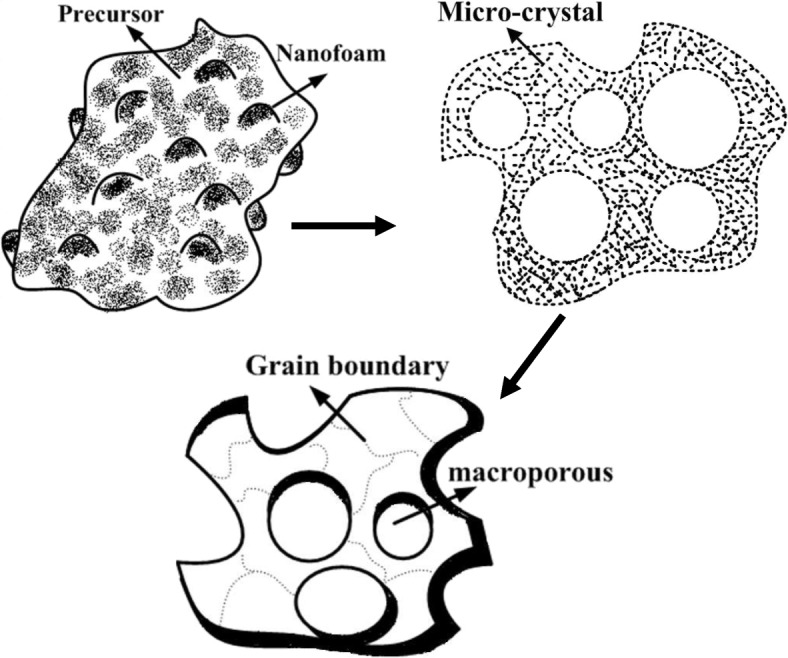
The schematic mechanism for the growth of La_3_Ga_5.5_Nb_0.5_O_14_ macroporous via a sol-gel route in our specially designed precursor solution

A proposed schematic growth mechanism of La_3_Ga_5.5_Nb_0.5_O_14_ macroporous based on the details provided is shown in Fig. 6. In this process, synthesized macroporous oxide forms crystals at 600 °C as shown in the schematic Fig. 5a. As mentioned, the precursor powder is at 600 °C, and it has nanofoams which are nanostructured porous materials with diameters less than 100 nm. These nanofoams are bulk nanoporous materials with enlarged form and very low thickness. The formation of nanofoams takes place because of heating in the presence of oxygen. At this stage, the precursor is in non-crystalline form. When the annealing temperature is increased to 700 °C, the bulky nanofoams will pop-out due to less thickness, and it leaves a hole of diameter less than 100 nm. These nanofoams are bulk nanoporous materials which are filled with either liquid or gas. In this case, these nanofoams are filled with gas either oxygen or carbon dioxide. At this stage, the precursor is in non-crystalline form. When the annealing temperature is increased to 700 °C, these nanofoams which are hollow inside will pop-out leaving a hole of diameter less than 100 nm. During the 600 to 700 °C annealing process, these nanofoams tend to grow size bigger and eventually form macroporous holes. At the same time, a lot of microcrystals are formed in an irregular order around the nanofoam holes. After the nanofoams collapsed and sintering occurred, the grain size will grow and inter-facial energy will be decreased [24] (Fig. 6).

When the annealing temperature is increased to 800 °C, the microcrystals form into crystal grains which are hard, and these grains are separated by the boundary grains. In this process, the collapse rate of nanofoams is directly proportional to the crystallization process. If the calcined temperatures are increased, it encourages the growth of the oxide crystals. The structure formed will be crystallized, and the macroporous holes will be formed which are of different sizes ranging from 50 to 100 nm.

**Fig. 7:**
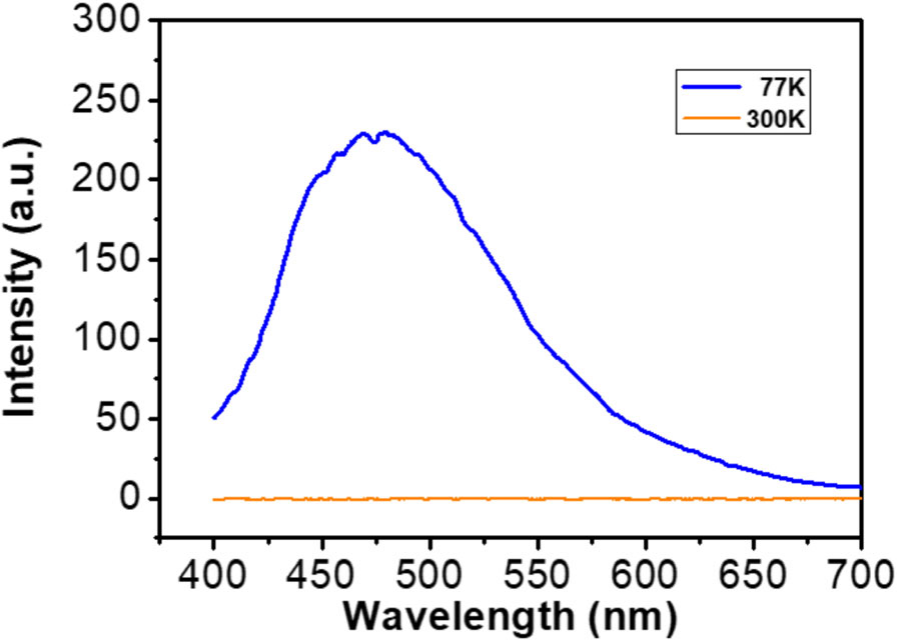
The room-temperature (300 K) and 77 K emission (*λ*_ex_ = 327 nm) spectra of pure La_3_Ga_5.5_Nb_0.5_O_14_ powders annealed at 800 °C

The emission spectra of the La_3_Ga_5.5_Nb_0.5_O_14_ samples at 77 K and 300 K are shown in Fig. 7. Photo-luminescence results show that the sample prepared at 77 K have exhibited emission spectra at 475 nm than the sample at room temperature 300 K. The ideal state temperature for the sample is 77 K, and at this temperature, there will be no thermal vibrations which affect the procedure. According to Blasse [25], there are two kinds of absorbing groups in niobate complexes which are [NbO_6_]^7−^ and [NbO_4_]^3−^. At 327 nm excitation spectra, there was only one peak appeared which corresponds to the [NbO_6_]^7−^ complex group. This indicates that the charge transfer happened in the bands of [NbO_6_]^7−^ in the La_3_Ga_5.5_Nb_0.5_O_14_ system. So here, the crystal structure of La_3_Ga_5.5_Nb_0.5_O_14_ might be built up by the edge-sharing of NbO_6_ trigonal prisms.

The PL emission spectra showed a strong blue emission spectra peak at 457 nm. Here, the luminescence effect depends on the Nb–O–Nb bonding where the conduction band consists of Nb^5+^ 4*d* orbitals and the valance band of O^2−^ 2*p* orbitals between the corner-sharing octahedral [26]. A strong temperature dependence of the emission peak was observed. The intensity of the emission peak reduced rapidly and nearly disappears when the temperature increased from 77 to 300 K. The quenching of the emission peak should be attributed to two reasons for thermal quenching effect in macroporous La3Ga5.5Nb0.5O14 ceramic. The one is that non-radiative transition results in the heat by transfer of energy to phonons in the lattices; another one is that the elections could be trapped by any possible defects in the lattices; and it is well known that the trap centers in niobate complexes, which could have an important quenching effect on luminescence [27, 28].

**Fig. 8:**
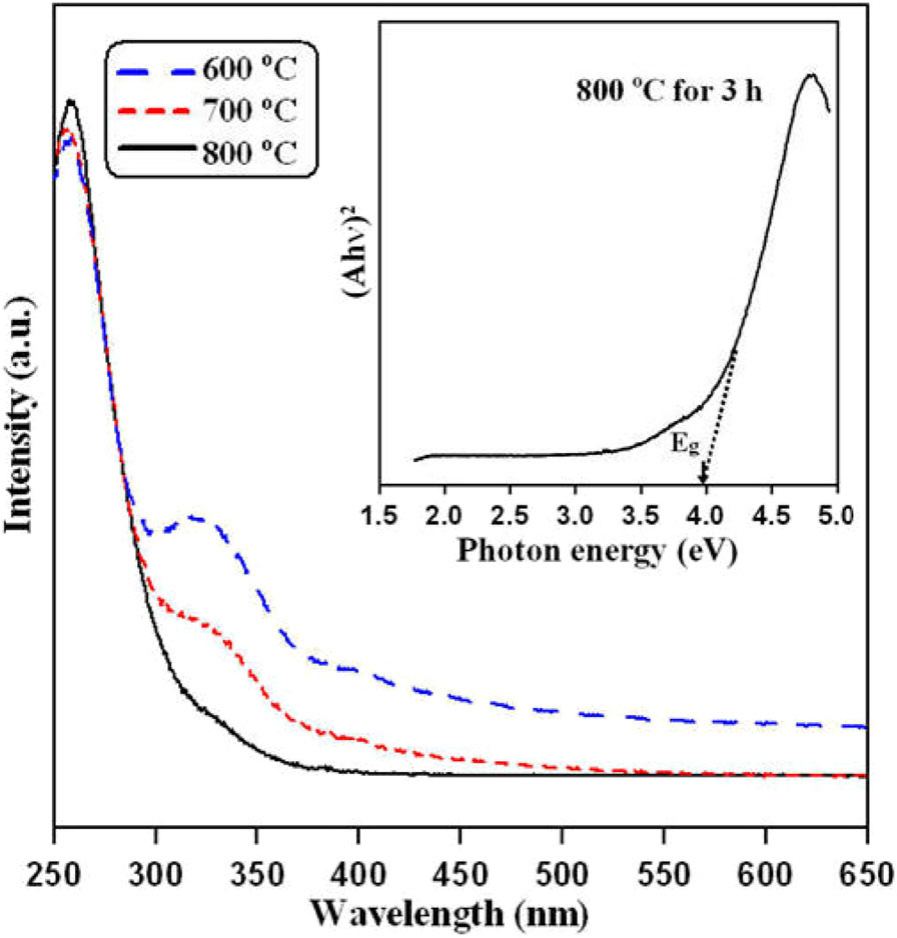
Absorption spectra of the La_3_Ga_5.5_Nb_0.5_O_14_ powders annealed at 600–800 °C for 3 h measured at room temperature. The inset is the behavior of optical absorption as the function of photon energy for La_3_Ga_5.5_Nb_0.5_O_14_ macroporous at 800 °C

UV-Vis absorption spectra of the La_3_Ga_5.5_Nb_0.5_O_14_ macroporous particles are measured, and band gap is estimated from the absorption spectra in Fig. 8. The absorption luminescence has a maximum intensity of 260 nm which corresponds with the excitation spectra. The absorbance in the vicinity of the onset due to the electronic transition for a given semiconductor is given by the following equation:

3 $$ \alpha =\frac{C{\left(\mathrm{h}\upnu -{E}_{\mathrm{g}}\right)}^{1/2}}{\mathrm{h}\upnu} $$

where *α* is the absorption coefficient, *C* is the constant, hν is the photon energy, and *E*_g_ is the band gap. The inset of Fig. 8 shows the relationship of (*α*hν)^2^ and hν*.* The inset in Fig. 8 shows the band gap of 3.95 eV. In the experiment, there are little bumps present at about 320 nm from 600 to 800 °C. These bumps indicate the presence of oxygen vacancy defect [29]. At 800 °C annealing temperature, the organic compound fiercely burnt very rapidly, and this consumed a great amount of oxygen. It is also noted that the defects are high at 600 °C, and with the increase in temperature, these vacancy defects are reduced.

## Conclusions

La_3_Ga_5.5_Nb_0.5_O_14_ macroporous polycrystal is prepared by a Pechini process using NbCl_5_, Ga(NO_3_)_3_, and La(NO_3)3_. This crystallized orthorhombic La_3_Ga_5.5_Nb_0.5_O_14_ can be obtained by the heat treatment process at 800 °C from XRD. The excitation wavelength is about 327 nm, and this is associated with charge transfer bands of Nb^5+^ and O^2−^ ions in a tetrahedral co-ordination. The photo-luminescence spectra shown that under 327 nm excitation spectra, a broad and blue emission peak is observed at 475 nm and this spectrum is originated from the [NbO_6_]^7−^ octahedra group. The visible light absorption edge of 800 °C sample was at 320 nm, which corresponded to the band gap energies of 3.95 eV.

## Data Availability

The authors declare that the materials and data are promptly available to readers without undue qualifications in material transfer agreements. All data generated in this study are included in this article.

## Abbreviations

BAW: Bulk acoustic wave
CA: Citric acid
CGC: Calciumgallogermanate
DTA: Differential thermal analysis
EDX: Energy dispersive X-ray analysis
*E*_g_: Band gap
EG: Ethylene glycol
FTIR: Fourier-transform infrared spectroscopy
hν: Photon energy
PL: Photoluminescence
SAW: Surface acoustic wave
TEM: Transmission electron microscopy
TGA: Thermogravimetry analysis
UV-vis: Ultraviolet-visible
XRD: X-ray powder diffraction
